# Supplementary material for: Brain-wide microstrokes affect the stability of memory circuits in the hippocampus
Source: Nat Commun. 2025 Apr 11;16:3462. doi: 10.1038/s41467-025-58688-4 (PMC11992252; doi:10.1038/s41467-025-58688-4)
Supplement: Supplementary file 1 — Supplementary information PDF [file 41467_2025_58688_MOESM1_ESM.pdf]

Supplementary information

for

Brain-wide microstrokes affect the stability of memory circuits in the hippocampus

Hendrik Heiser[1,2], Filippo Kiessler[3], Adrian Roggenbach[1,2], Victor Ibanez[1,2], Martin Wieckhorst[1,2], Fritjof Helmchen[1,2,4], Julijana Gjorgjieva[3], Anna-Sophia Wahl[1,2,5,6*]

Supplementary Tables S1, S2

Supplementary Figures S1-S8

Table S1: Statistical tests, post-hoc tests (if applicable) and exact p-values (3 decimal places) of significant comparisons used in the figure panels. ANOVA = analysis of variance; RM = repeated measures; GG = Greenhouse-Geisser correction; REML = restricted maximum likelihood estimation.

Panel	Statistical test	Post-hoc test/multiple comparisons correction	Comparison	P-value
1F-G	Pearson correlation & linear regression	n.a.		
1J-L	RM two-way ANOVA with GG	Tukey-Kramer	1J – Early post: Sham vs. Stroke	P=0.005
			1J – Late post: Sham vs. Stroke	P=0.025
			1L – Late post: Sham vs. Stroke	P=0.046
2B	Wilcoxon matched-pairs signed-rank test	n.a.	PC-PC vs. NC-NC	P<0.001
	One-sample t-tests (H_0 = 0)	Bonferroni	PC-PC	P<0.001
			NC-PC	P=0.004
2C left	RM mixed-effects model with REML	Bonferroni	Sham: PC-PC vs. NC-NC	P<0.001
			PC-PC: Sham vs. Stroke	P<0.001
	One-sample t-tests (H_0 = 0)	Bonferroni	Sham: PC-PC	P<0.001
			Sham: NC-PC	P=0.008
2C right	RM mixed-effects model with REML	Bonferroni	Sham: PC-PC vs. NC-NC	P<0.001
			Stroke: PC-PC vs. NC-NC	P<0.001
	One-sample t-tests (H_0 = 0)	Bonferroni	Sham: PC-PC	P<0.001
			Sham: NC-PC	P=0.016
			Stroke: PC-PC	P=0.004
			Stroke: NC-PC	P=0.025
2D	RM two-way ANOVA	Bonferroni	sPC-sPC: Sham vs. Stroke	P=0.004
			sPC-NC: Sham vs. Stroke	P<0.001
	One-sample t-tests (H_0 = 0)	Bonferroni	Sham: sPC-sPC	P<0.001
			Sham: sPC-NC	P<0.001
			Sham: uPC-sPC	P=0.027
			Sham: uPC-NC	P=0.004
			Sham: NC-sPC	P=0.012
			Sham: NC-NC	P=0.009
3A	RM mixed-effects model with REML	Tukey-Kramer	D3 – No-Recovery vs. Sham	P<0.001
			D3 – Recovery vs. Sham	P<0.001
			D6 – No-Recovery vs. Sham	P=0.002
			D9 – No-Recovery vs. Recovery	P=0.013
			D9 – No-Recovery vs. Sham	P=0.004
			D15 – No-Recovery vs. Recovery	P=0.039
			D18 – No-Recovery vs. Recovery	P=0.020
			D18 – No-Recovery vs. Sham	P<0.001
3B	RM two-way ANOVA with GG	Tukey-Kramer	Sham: Healthy vs. Late post	P=0.020
			Sham: Early post vs. Late post	P=0.002
			Early post: No-Recovery vs. Sham	P=0.005

			Late post: No-Recovery vs. Sham	P=0.009
3D	RM two-way ANOVA with GG	Tukey-Kramer	No Recovery: Healthy vs. Early post	P=0.007
			No Recovery: Healthy vs. Late post	P=0.037
			Recovery: Early post vs. Late post	P=0.038
			Early post: No-Recovery vs. Sham	P<0.001
			Early post: Recovery vs. Sham	P=0.003
			Late post: No-Recovery vs. Sham	P=0.045
3E	RM two-way ANOVA with GG	Tukey-Kramer	No Recovery: Healthy vs. Early post	P=0.009
			Early post: No-Recovery vs. Sham	P=0.002
			Recovery: Early post vs. Late post	P=0.045
3F	RM two-way ANOVA with GG	Tukey-Kramer	Sham: Healthy vs. Late post	P=0.008
			Sham: Early post vs. Late post	P=0.013
			No-Recovery: Healthy vs. Early post	P=0.002
			No-Recovery: Healthy vs. Early post	P=0.009
			Early post: No-Recovery vs. Sham	P<0.001
			Late post: No-Recovery vs. Sham	P=0.006
	One-sample t-tests (H_0 = 0.0125)	Bonferroni	Sham: Healthy	P<0.001
			Sham: Early post	P<0.001
			Sham: Late post	P<0.001
			No-Recovery: Healthy	P<0.001
4B	group-wise one-way ANOVA with GG		Sham (Group x Time)	P=0.002
			Recovery (Group x Time)	P=0.016
	RM two-way ANOVA with GG	Tukey-Kramer	Late-Late: No-Recovery vs. Sham	P=0.003
			Late-Late: No-Recovery vs. Sham	P=0.030
4C	RM two-way ANOVA with GG	Tukey-Kramer	Sham: Healthy-Healthy vs. Late-Late	P=0.018
			Recovery: Healthy-Stroke vs. Late-Late	P=0.039
			No-Recovery: Healthy-Healthy vs. Healthy-Stroke	P=0.004
			No-Recovery: Early-Early vs. Late-Late	P=0.034
			Healthy-Stroke: No-Recovery vs. Sham	P<0.001
			Healthy-Stroke: Recovery vs. Sham	P=0.010
			Early-Early: No-Recovery vs. Sham	P<0.001
4G	RM two-way ANOVA with GG	Tukey-Kramer	Sham: Healthy-Healthy vs. Healthy-Stroke	P<0.001
			Sham: Healthy-Stroke vs. Stroke-Stroke	P<0.001
			No-Recovery: Healthy-Healthy vs. Healthy-Stroke	P=0.023
			No-Recovery: Healthy-Healthy vs. Stroke-Stroke	P=0.028
			Healthy-Stroke: No-Recovery vs. Sham	P<0.001
			Stroke-Stroke: No-Recovery vs. Sham	P<0.001
5B	RM two-way ANOVA with GG	Tukey-Kramer		
5D	RM two-way ANOVA with GG	Tukey-Kramer	Early post: No-Recovery vs. Sham	P=0.048
			Recovery: Early post vs. Late post	P=0.025
5E	RM two-way ANOVA with GG	Tukey-Kramer	No-Recovery: Healthy vs. Early post	P=0.026
			Recovery: Healthy vs. Early post	P=0.001
			Recovery: Early post vs. Late post	P=0.021
			Early post: No-Recovery vs. Sham	P=0.001
5G	RM two-way	Tukey-Kramer	Healthy – Sham: Far vs. In	P=0.029

	ANOVA with GG			
			Late post – Recovery: Far vs. Close	P<0.001
			Late post – Recovery: Close vs. In	P=0.003
			Late post – Close: Recovery vs. Sham	P=0.002
S1C	Kruskal-Wallis	Dunn	Sham vs. Recovery	P=0.049
			Sham vs. No Recovery	P=0.025
S1D	Mann-Whitney test	n.a.	Recovery vs. No Recovery	P<0.001
S1E	Spearman correlation & linear regression	n.a.	Neocortex: Spheres vs. Lesion volume	P=0.016
			Thalamus: Spheres vs. Lesion volume	P<0.001
S1F	Generalized linear model	n.a.		
S1G	Pearson correlation & linear regression	n.a.		
S1H	Generalized linear model	n.a.		
S1I	Ordinary Least Squares regression	n.a.		
S2A	RM mixed-effects model with REML	Bonferroni	Healthy vs. Day 2	P=0.018
S2B	RM one-way ANOVA with GG	Bonferroni	Healthy vs. Day 2	P=0.034
S2C	RM two-way ANOVA	Fisher's LSD with Bonferroni	Stroke: Sham vs. Stroke	P=0.024
S2 D-F	RM two-way ANOVA with GG	Bonferroni	Sham: Healthy vs. Early post	P=0.001
			Recovery: Healthy vs. Early post	P=0.048
			Recovery: Early post vs. Late post	P=0.001
S3	RM two-way ANOVA	Bonferroni		
	One-sample t-test (H_0 = 0)	Bonferroni	Sham: NC-NC	P=0.005
			Sham: NC-sPC	P=0.014
			Sham: uPC-NC	P=0.026
			Sham: uPC-uPC	P=0.018
			Sham: sPC-NC	P<0.001
			Sham: sPC-uPC	P=0.045
			Sham: sPC-sPC	P<0.001
			Stroke: NC-NC	P=0.027
S4B	RM two-way ANOVA with GG	Tukey-Kramer	No-Recovery: Healthy vs. Healthy-Stroke	P=0.011
			Healthy-Stroke: No-Recovery vs. Sham	P<0.001
			Early-Early: No-Recovery vs. Sham	P<0.001
			Late-Late: No-Recovery vs. Sham	P=0.013
			Late-Late: No-Recovery vs. Recovery	P=0.011
S4C	Pearson correlation & linear regression	n.a.		

S5A	Pearson correlation	n.a.		
S5B	RM two-way ANOVA with GG	Tukey-Kramer	Early post: Sham vs. No-Recovery	P=0.015
			Early post: Sham vs. Recovery	P=0.035
			Late post: Sham vs. No-Recovery	P=0.033
S6A	RM two-way ANOVA with GG	Tukey-Kramer		
S6B	Pearson correlation & linear regression	n.a.		
S8A	Mann-Whitney test	n.a.		
S8B	RM mixed-effects model with REML	Šídák		
S8C-E	RM two-way ANOVA with GG	Šídák		

Table S2: General information of animals used in the experiments. “ID” represents the unique internal ID of each animal. “Recovery” and “No Recovery” outcome groups were part of the “Stroke” group. The list in “Imaged neurons” indicate the total number of neurons imaged during each imaging session for each mouse.

ID	Strain	Sex	Group	Imaged neurons
33	WT	M	Sham	[407, 385, 426, 382, 347, 312, 379, 352, 359, 307]
41	Snap25-GCaMP6f	F	No Recovery	[1313, 1116, 1048, 1321, 1282, 948, 880, 1298, 776, 873, 905, 952]
63	WT	F	No Recovery	[89, 115, 105, 96, 113, 56, 54, 40, 36, 88, 160, 210, 168, 199]
69	WT	M	No Recovery	[812, 880, 1220, 679, 936, 1092, 945, 1092, 759, 1065, 1251, 1586, 1323, 1230, 901]
83	WT	F	Sham	[564, 585, 545, 547, 577, 655, 543, 490, 532, 485, 661, 534, 570, 781, 702, 628, 609, 654, 636]
85	WT	F	Recovery	[537, 687, 653, 423, 524, 443, 424, 653, 688, 728, 615, 618, 491, 699, 754, 444]
86	WT	F	Recovery	[682, 773, 795, 804, 717, 809, 726, 785, 708, 689, 708, 733, 708, 713, 727, 783]
89	WT	F	Recovery	[163, 170, 167, 152, 150, 169, 116, 103, 88, 117, 155, 65, 96, 168, 160, 166, 176, 185, 199]
90	WT	F	Recovery	[769, 711, 605, 767, 651, 706, 480, 845, 765, 753, 937, 573, 739, 663, 770, 747]
91	Thy1-GCaMP6f	M	Sham	[1197, 1224, 1143, 1183, 1298, 1113, 1082, 1176, 1252, 1204, 999, 1650, 1258, 1620, 1278]
93	Thy1-GCaMP6f	M	Sham	[1372, 1264, 1272, 1520, 1482, 1440, 1408, 1485, 1452, 1530, 1592, 1497, 1538, 1447, 1515]
95	Thy1-GCaMP6f	F	Sham	[550, 710, 678, 712, 624, 755, 860, 690, 596, 781, 884, 811, 991, 1098, 1028, 767, 951, 821]
108	Thy1-GCaMP6f	F	Sham	[941, 1241, 688, 958, 863, 1097, 848, 1251, 1264, 1403, 1249, 947, 1430, 1023, 1236]
110	Thy1-GCaMP6f	F	No Recovery	[407, 460, 486, 411, 443, 555, 544, 423, 491, 346, 620, 471, 534, 422, 483]
111	Thy1-GCaMP6f	F	Sham	[415, 508, 460, 512, 622, 659, 631, 663, 572, 779, 707, 849, 711, 569, 737]
113	Thy1-GCaMP6f	F	Recovery	[1076, 1006, 994, 747, 738, 515, 719, 769, 788, 857, 900, 809, 609, 821, 640]
114	Thy1-GCaMP6f	F	Sham	[634, 825, 751, 601, 506, 561, 523, 627, 701, 805, 703, 703, 557, 591]
115	Thy1-GCaMP6f	F	Sham	[380, 415, 421, 338, 298, 331, 302, 443, 257, 393, 434, 464, 470, 432, 396]
116	Thy1-GCaMP6f	M	Sham	[947, 931, 860, 849, 894, 872, 734, 969, 969, 652, 1051, 978, 1006, 868, 928]
122	Thy1-GCaMP6f	F	Sham	[439, 413, 398, 434, 390, 401, 391, 451, 455, 486, 520, 476, 342, 414, 432]

Figure S1

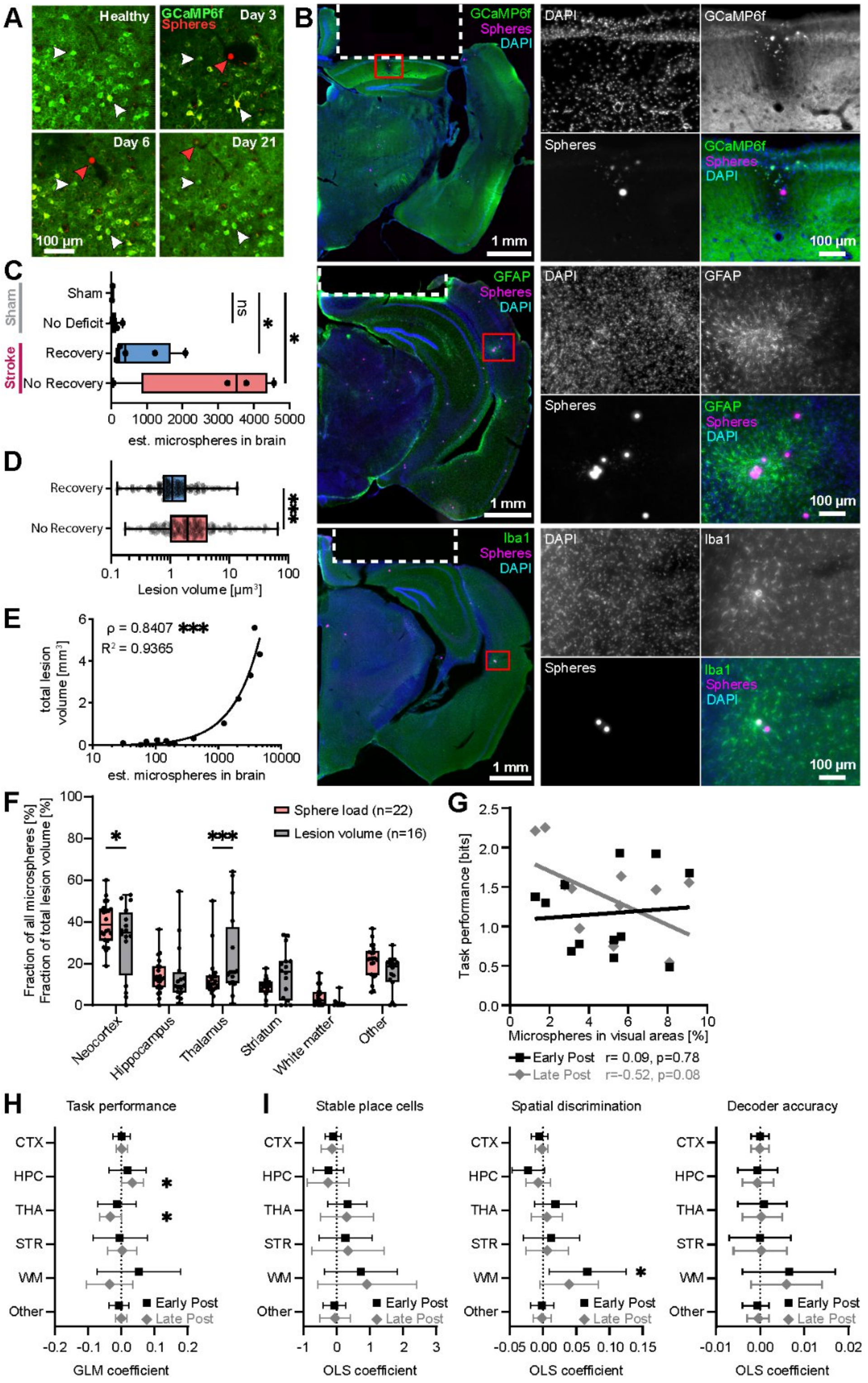

Figure S1: Microsphere injections cause hemisphere-wide lesions. A. Section of a field of view of a hippocampal CA1 network under the two-photon microscope at four time points. White arrows show the same neurons in each image. A red fluorescent

microsphere in the tissue is visible after microsphere injection (red arrow). The black region around the sphere at day 3 is hypothesized to be microglia engulfing the foreign object and absorbing GCaMP6f fluorescence of the surrounding tissue. The sphere moves slightly with respect to the adjacent neurons over time, and does not cause visible tissue damage. **B.** Exemplary widefield images of microspheres and resulting lesions in stainings for neurons (top row, Thy-1 transgenic GCaMP6f expression), astrocytes (middle row, α-GFAP antibody), and microglia (bottom row, α-Iba1 antibody). Microspheres and lesion-caused autofluorescence is visible in the red (Cy5) channel. Left column shows whole hemispheres (scale bar = 1 mm), the right column shows high-magnification sections of microspheres and surrounding tissue (scale bar = 100 µm). Some, but not all spheres cause tissue damage, detectable by reduced GCaMP6f signal (top) as well as increased astrocyte (middle) and microglia (bottom) activity. Dashed lines in the overview images (left) indicate location of the hippocampal window implant. **C.** Extrapolated counts of microspheres in the whole brains of injected animals across groups (Sham, n=2; No Deficit, n=9; Recovery, n=5; No-Recovery, n=4). Statistics assess by a Kruskal-Wallis test with Dunn's multiple comparisons test. **D**. Estimated volume of individual lesions in Recovery (n=175 lesions) and No-Recovery (n=219 lesions) animals. “No Recovery” animals have significantly larger lesions than “Recovery” animals. **E.** Total lesion volume correlates strongly with number of detected microspheres. Microsphere loads show a skewed distribution with most mice having few, and few mice having many microspheres in their brains. Line shows the best fit of a linear regression model (n=13 mice). **F.** Fraction of spheres (n=22 mice) and lesion volume (n=16 mice) across different brain regions following the Allen Atlas nomenclature. “Other” are regions not included in the other groups: cerebellum, midbrain, hindbrain, pallidum, cortical subplate, hypothalamus, olfactory areas. Statistics assessed by a subject-matched mixed-effects model with Bonferroni multiple comparisons test. **G**. The relative amount of microspheres in visual areas (“VIS” in reference to the Allen Atlas nomenclature) does not significantly correlate with task performance during early or late poststroke periods, suggesting no direct link between damage to the visual system and task performance. **H.** Influence of microsphere counts in different brain regions on the relative VR task performance change compared to the healthy baseline performance during early and late post-stroke, evaluated by generalized linear models (Gamma distribution, identity link). **I.** Influence of microsphere counts in different brain regions on metrics of neural coding, evaluated by Ordinary Least Squares regression. Brain regions are defined identically to panel F. Error bars show 95% confidence intervals (n = 18). Boxplots are drawn with the box extending from the 25th to 75th percentiles, with the center line at the median. Whiskers reach to the minimum and maximum values of the distribution. Asterisks indicate significances: *p<0.05, **p<0.01, ***p<0.001.

Figure S2

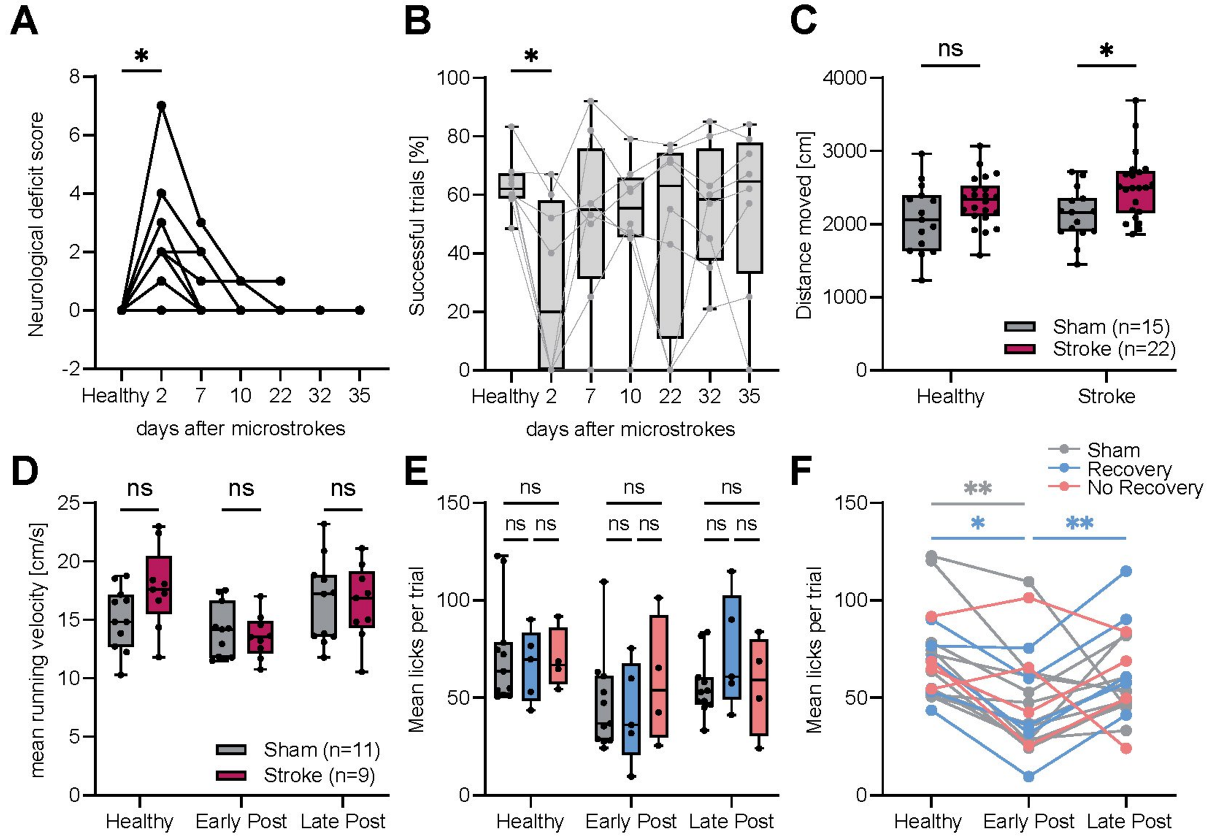

Figure S2: Motor functions remain largely unaffected by microstrokes. A. Neurological deficit scores[64] measured before and after microstrokes (n=13 mice). Mice showed a significant deficit only on the first session two days after microsphere injection. **B.** Percentage of successful trials in a forelimb motor task testing strength and grasping skills measured before and after microstrokes. Mice showed a significant deficit only on the first session two days after microstrokes. **C.** Total distance moved in an open field of stroke (n=22) and sham (n=15) mice, tested before and within the first week after microstroke surgery. Stroke mice were more active than sham mice in the post-stroke session (Sham: 2472±92 cm, Stroke: 2506±96 cm, p=0.006). **D.** Mean running velocity of Stroke (n=9) and Sham (n=11) mice in the VR corridor during the experiment. Microstrokes did not affect running speed, as sham and stroke mice did not show significant differences before or after surgery. **E.** Mean number of licks per trial in the VR corridor across experimental groups (Sham, n=11; Recovery, n=5; No-Recovery, n=4) and stages. Microstrokes did not affect licking behavior, as sham and stroke mice did not show significant differences before or after surgery. **F**. Temporal dynamic of average licks per trial in the three outcome groups (Sham, n=11; Recovery, n=5; No-Recovery, n=4) across experimental phases. Measured performance changes are likely not purely due to a lack of task engagement, as “No Recovery” mice with the largest performance deficit show no change in the lick rate. Statistics were evaluated using two-way repeated-

measures ANOVA with Greenhouse-Geisser correction and Tukey-Kramer multiple comparisons test. Boxplots are drawn with the box extending from the 25th to 75th percentiles, with the center line at the median. Whiskers reach to the minimum and maximum values of the distribution. Asterisks indicate significances: *$p<0.05$, **$p<0.01$, ***$p<0.001$.

Figure S3

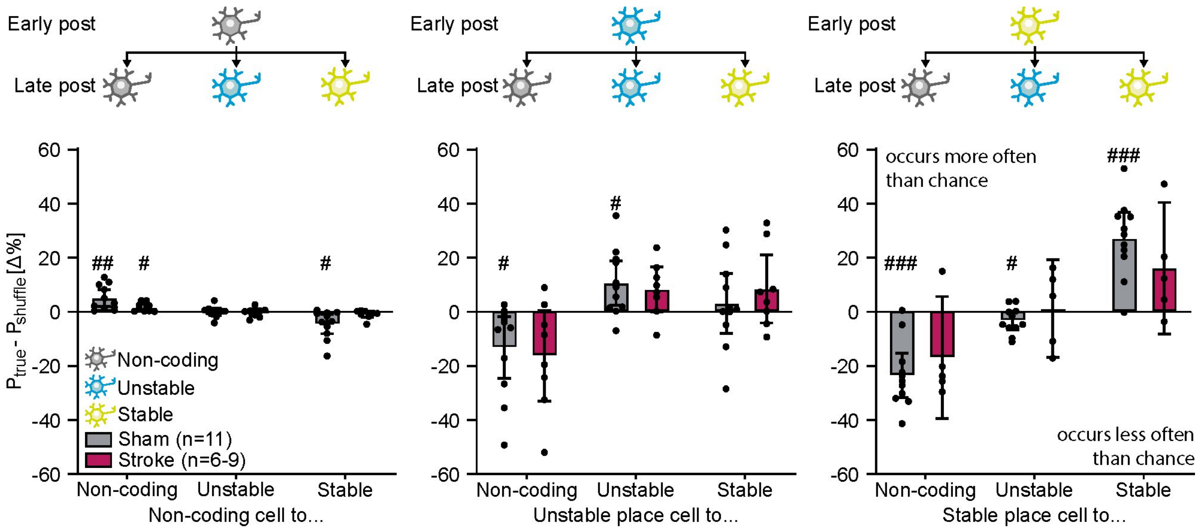

Figure S3: Microstroke-induced loss of functional imprinting for stable place cells is partially reversed during late post-stroke. Observed probabilities of non-coding cells (grey), unstable (blue) and stable (yellow) place cells to maintain or switch their functional class from the early to late post-stroke phase in Stroke (n=6-9) and Sham (n=11) mice. Data are presented as mean values +/- SD. Asterisks indicate significances of two-way repeated-measures ANOVA with Bonferroni multiple comparisons test. Hash symbols indicate significances of one-sample t-tests with Bonferroni correction to chance level (ΔP=0). Significance levels: */# p<0.05, **/## p<0.01, ***/### p<0.001.

Figure S4

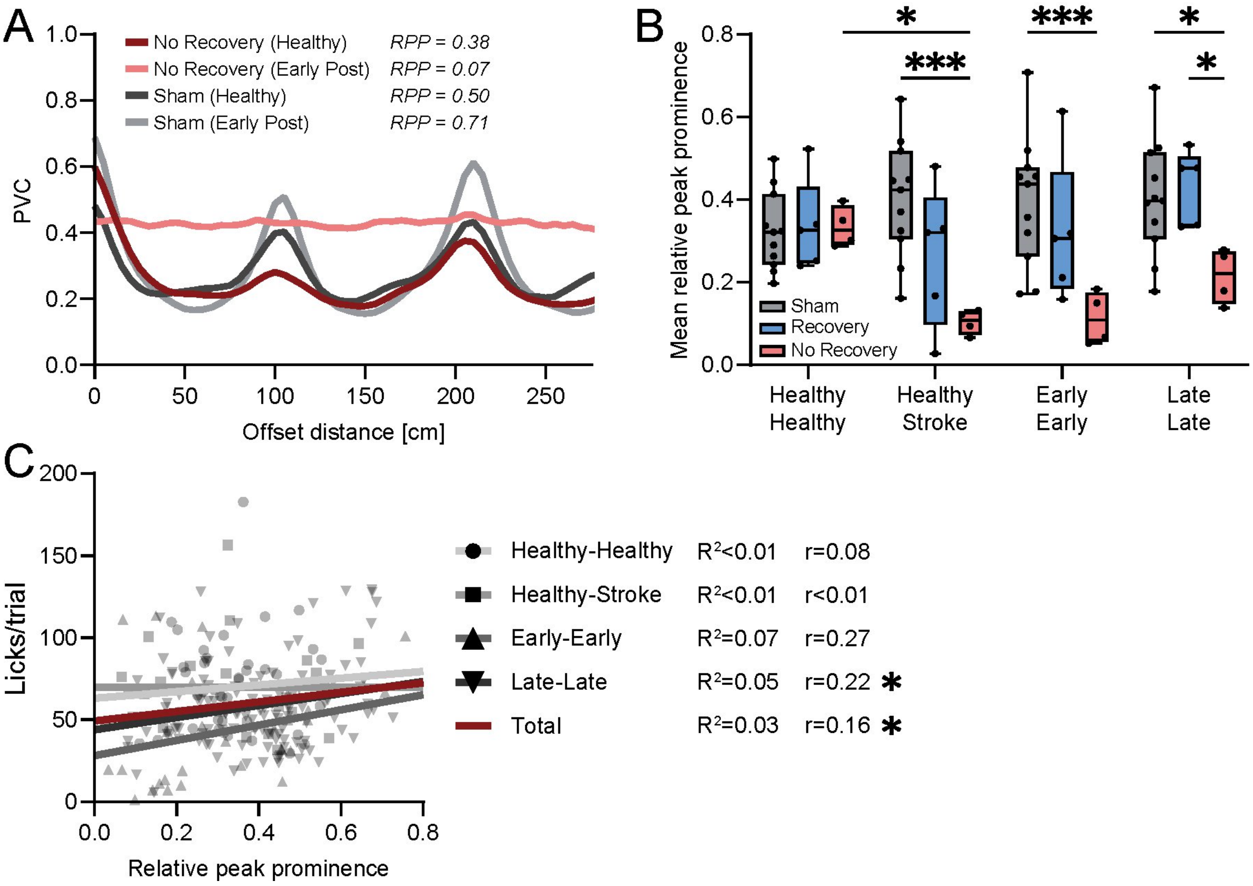

Figure S4. PVC curve flatness. A. Exemplary individual PVC curves from a "No Recovery" and "Sham" animal during the healthy and early post-stroke periods. The "flatness" of the curves is quantified by the "relative peak prominence" (RPP). **B**. Quantification of relative peak prominence across experimental groups (Sham, n=11; Recovery, n=5; No-Recovery, n=4) and stages. RPP is not significantly different between groups before stroke, and follows a similar pattern as other PVC metrics (see figure 4). **C**. Correlation of relative peak prominence and average licks per trial across session pairs within experimental phases (Healthy-Healthy, n=44; Healthy-Stroke, n=21; Early-Early, n=28; Late-Late, n=122; Total, n=215). Only the dataset consisting of late post-stroke sessions as well as the whole dataset is significantly correlated, but with low goodness of fit ($R^2 < 0.1$), suggesting that the loss of periodicity does not simply reflect changes in lick rates. Boxplots are drawn with the box extending from the 25th to 75th percentiles, with the center line at the median. Whiskers reach to the minimum and maximum values of the distribution. Asterisks indicate significances: *p<0.05, **p<0.01, ***p<0.001.

Figure S5

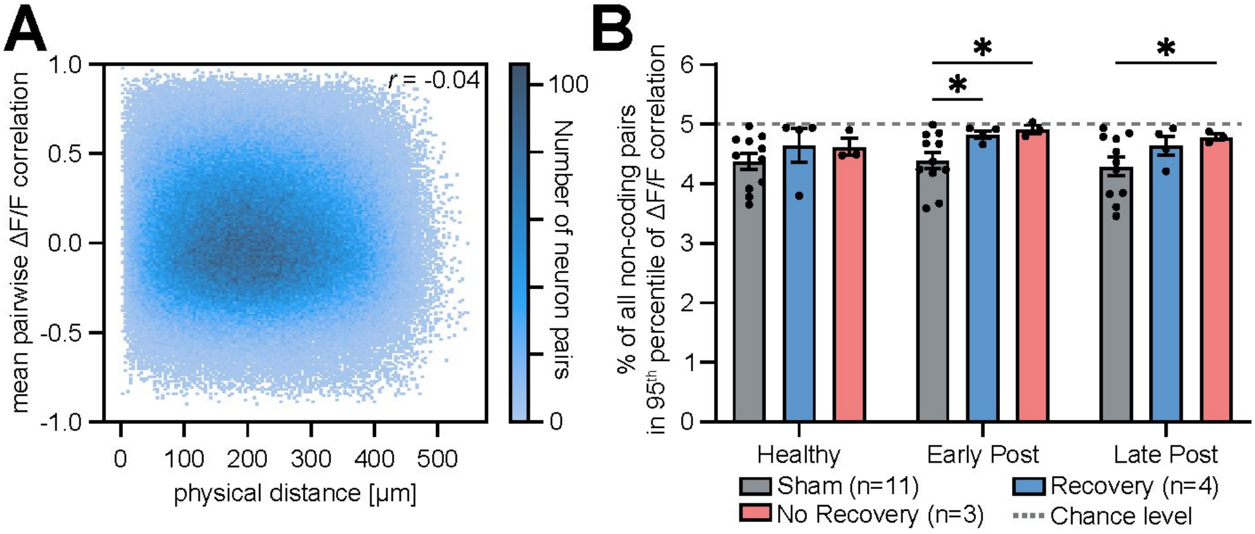

Figure S5: Functional synchronicity. A. Color-coded histogram of the physical distance between two cells and their average pairwise ΔF/F correlation across all animals (n=20). Darker colors indicate more cell pairs. No strong relation (Pearson correlation coefficient *r* = -0.04) between physical distance and ΔF/F correlation is visible. **B**. Percentage of non-coding cell pairs detected within the 95th percentile of highly correlated cell pairs across experimental groups (Sham, n=11; Recovery, n=4; No-Recovery, n=3) and phases. Dashed line indicates chance level of a uniform distribution (5%). In B, data are presented as mean values +/- SEM. Asterisks indicate significances: *p<0.05, **p<0.01, ***p<0.001.

Figure S6

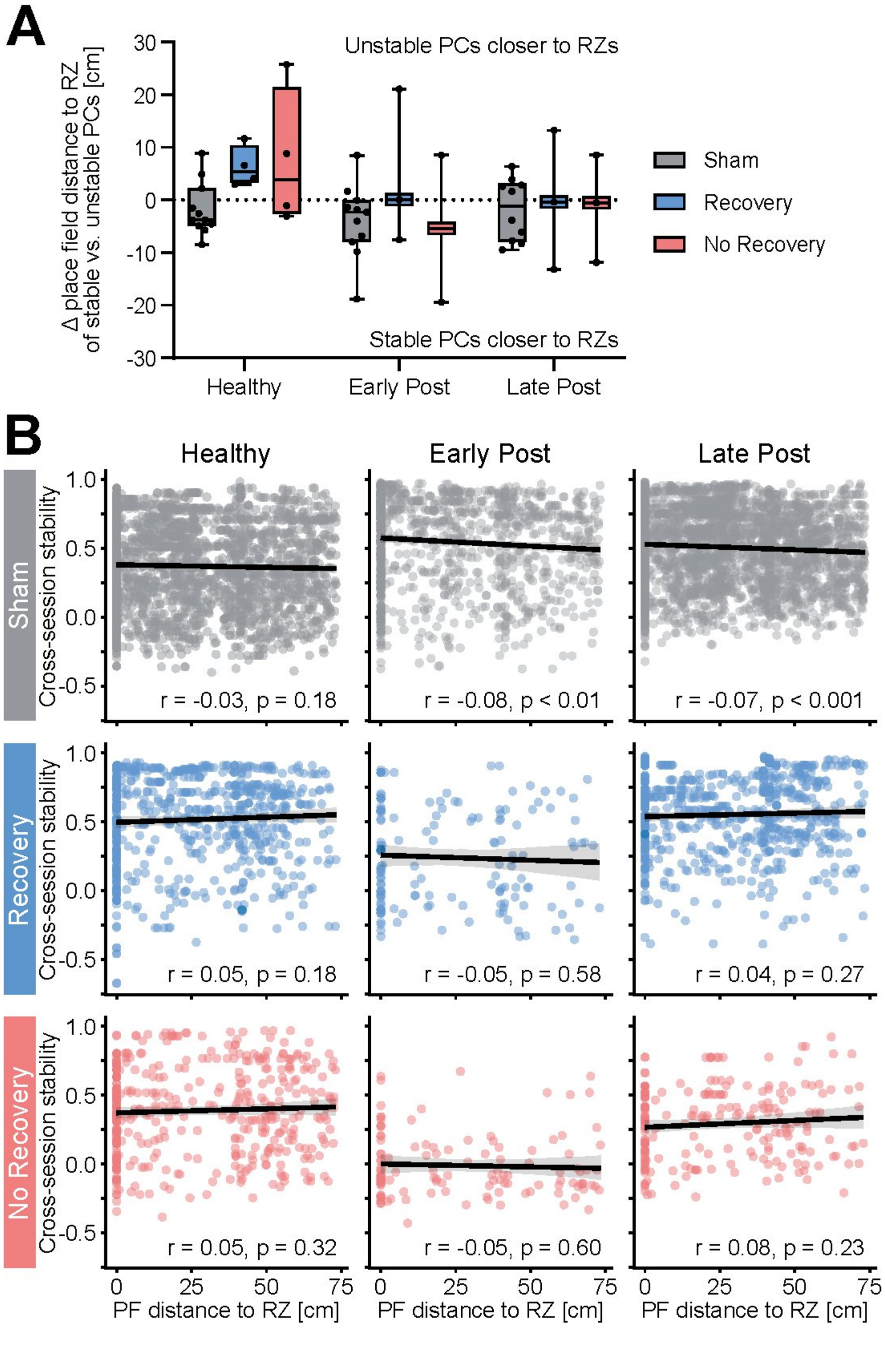

Figure S6: Distance to reward only weakly affects place cell stability. A. Difference in distance of place fields to the next reward zone across experimental groups (Sham, n=11; Recovery, n=5; No-Recovery, n=4) and stages. No dataset is significantly different from 0 (Wilcoxon signed-rank tests with Bonferroni correction). **B**. Place field distance to the next reward zone of individual place cells pooled for animals of the same experimental group and phase and plotted against the cross-session stability of the neuron. Only the "Sham" group shows a weak but significant negative correlation between place field

distance and cross-session correlation. Shaded area around linear regression line indicates 95% confidence interval. Boxplots are drawn with the box extending from the 25th to 75th percentiles, with the center line at the median. Whiskers reach to the minimum and maximum values of the distribution.

Figure S7

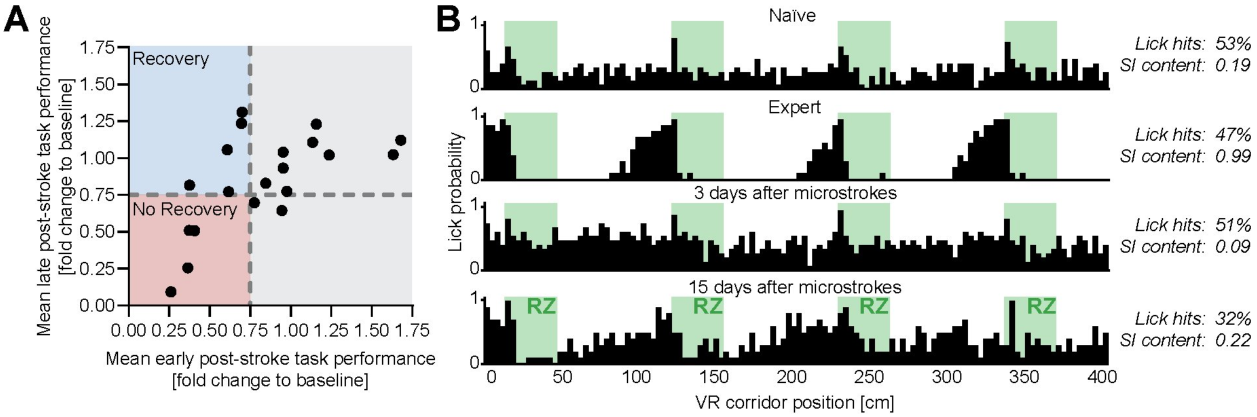

Figure S7. Diversity of behavioral patterns. A. Scatter plot of the average relative task performance during early and late poststroke of all animals (n=20). Mice with a relative performance of <75% only during early poststroke were categorized as "Recovery", animals with a relative performance of <75% during both early and late poststroke were labeled as "No Recovery". **B**. Lick histograms of an exemplary animal that displayed extensive anticipatory licking during the expert phase. The SI-based performance metric reflects the focused licking pattern also with this strategy, while a simpler metric of the percentage of licks within reward zones (lick hits) could not accurately represent the performance of the animal.

Figure S8

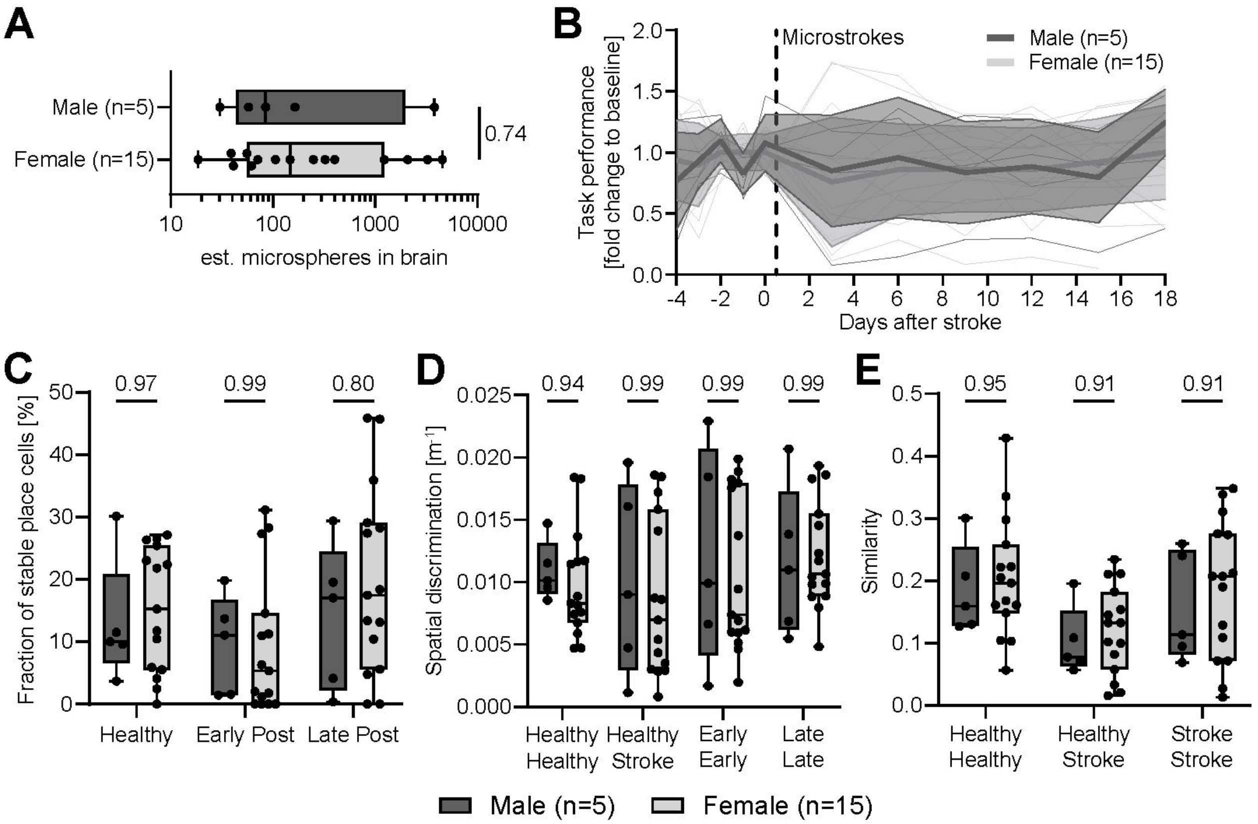

Figure S8. Sex does not affect microstroke outcome. A. Extrapolated counts of microspheres in the whole brains of injected male (n=5) and female (n=15) animals. **B**. VR performance after stroke relative to the healthy baseline of male and female mice. A mixed-effects model with the Greenhouse-Geisser correction and Tukey-Kramer multiple comparisons test revealed no significant difference between sexes ($F_{Sex}(1, 18)=0.062$, $p=0.81$) or across time ($F_{Sex\ x\ Time}(11, 191)=0.749$, $p=0.69$). Data are presented as mean values +/- SD. **C.** Percentages of stable place cells out of all imaged neurons in male and female mice. **D.** The absolute initial slope of PVC curves, which represents spatial precision in neural location coding, with higher values (steeper slopes) indicating higher precision, in male and female mice. **E.** Mean Cosine similarity of functional correlations of spatially binned activity in male and female mice. P-values were computed in the whole figure by two-way repeated-measures ANOVA with Bonferroni multiple comparisons test. Boxplots are drawn with the box extending from the 25th to 75th percentiles, with the center line at the median. Whiskers reach to the minimum and maximum values of the distribution.
